# Substance Use among Homeless Reproductive Age People in Southern Ethiopia

**DOI:** 10.1155/2021/8272986

**Published:** 2021-03-15

**Authors:** Negash Wakgari, Terefe Woyo, Emnet Kebede, Hirut Gemeda, Wakgari Binu, Gonfa Moti

**Affiliations:** ^1^Department of Midwifery, Ambo University, Ambo, Ethiopia; ^2^Department of Midwifery, Hawassa University, Hawassa, Ethiopia; ^3^School of Public Health, Wolaita Soddo University, Wolaita, Ethiopia; ^4^Ambo University Referral Hospital, Ambo, Ethiopia

## Abstract

**Introduction:**

Substance use by homeless reproductive age people may result to anxiety, involvement of risky sexual behaviors, and increasing the likelihood of unwanted pregnancy and sexually transmitted diseases (STDs). Therefore, this study assessed the magnitude of alcohol use, sexual intercourse after alcohol use, and its associated factors among homeless reproductive age people in southern Ethiopia.

**Methods:**

Community-based cross-sectional study design was conducted among homeless reproductive age people. The snowball sampling technique was used to recruit 842 participants. Pretested and structured interviewer-administered questionnaire was used to collect the data. Data were entered into Epidata version 3.1 and exported to SPSS version 23 for analysis. Binary logistic regression was used to determine the association of independent variables with the outcome variables. Odds ratio with their 95% confidence interval and *P* value was used to identify the significant variables.

**Results:**

More than half 423 (53.2%) of the respondents had taken a drink that contains alcohol in the last one year of the study period. Out of 324 khat chewers, 190 (58.64%) had sex after chewing khat. More than one-thirds, 323(38.4%) homeless people were smoking cigarette during the study period. Factors associated with alcohol use were age 19-25 years (AOR: 0.49; CI: 0.34, 0.72), ≥26 years (AOR: 0.40; CI: 0.25, 0.65), level of education (AOR: 0.61; CI: 0.39, 0.94), place of residence: major urban (AOR: 0.31; CI: 0.18, 0.51), small town (AOR: 0.38; CI: 0.23, 0.63), ever heard about STDs (AOR: 0.14; CI: 0.07, 027), and being a cigarette smoker (AOR: 2.67; CI: 1.94, 3.71).

**Conclusions:**

In this study, significant percentage of respondents had taken a drink that contains alcohol. Age, level of education, place of residence, ever heard about STDs, and smoking cigarette were variables significantly associated with alcohol use. Awareness creation on the effect and outcome of substance use is recommended.

## 1. Introduction

The health effect of substance use among the general population is well documented [1–5]. A multiple use of substances such as tobacco, alcohol, marijuana, and khat is reported [2, 5]. Despite the awareness of its ill effect on the health, the severity of alcohol consumption among adolescents is highly prevalent [3, 6–9]. This would be much more problematic among homeless people due to their lifestyle and economic dependency [6, 10–13].

People who sleep on streets, main roads, railway terminals, bus stations, parks, religious centers, construction sites, and other public places are defined as homeless people [14]. Alcohol consumption by the street-dwelling population may result to anxiety, depression, stressful life events, relative abstinence, and low self-esteem [15, 16]. It may also lead the street-dwelling reproductive age group [15-49 years] to the involvement of risky sexual behaviors and increasing the likelihood of unwanted pregnancy, STDs, and its consequence [6, 17, 18]. In addition, sexual intercourse after alcohol use is linked to inconsistent condom use, commercial sex, and rape [19, 20]. Moreover, both executing a crime and being a victim of an offence are also associated with alcohol use [21].

The proportion of alcohol use and its associated factors varies from region to region based on the context and sociodemographic characteristics of the street-dwelling people. For instance, previous studies reported 38% in Texas [10], 40% in Toronto [22], 35% in Egypt [23], 63.5% in Democratic Republic of Congo [24], and 64% alcohol use among street-dwelling people in Addis Ababa, Ethiopia [18].

Factors such as gender, age, duration of stay on the street, and peer influence were reported in previous studies as the major triggers for substance use [12, 13, 18, 24–29]. Though there are some studies conducted on substance abuse, risky sexual behavior, sexual, and reproductive health status of street children and youths [17, 18], none of them focused specifically on alcohol consumption, sexual intercourse after alcohol use, and its associated factors among homeless reproductive age people [15-49 years] in the study setting. Similarly, few of the previous studies done in the study area were focused on homeless women and children [30–33] and fail to notice sexual intercourse after alcohol use among both sexes in small towns of Ethiopia considering the differences in sociodemographic characteristics, culture, and awareness about the substances uses across the country. Therefore, this study assessed the magnitude of alcohol use, sexual intercourse after alcohol use, and its associated factors among homeless reproductive age people in southern Ethiopia.

## 2. Materials and Methods

### 2.1. Study Setting, Design, and Population

Community-based cross-sectional study design was conducted among homeless reproductive age people in southern Ethiopia. Hawassa; Arba Minch, Soddo, Dilla, Hosanna, Durame, Jinka, Werabe, Welkite, Segen, Yergalem, Bench, Tercha, Masha, and Mizan Aman are major cities in the southern Ethiopia. All homeless reproductive age people [15-49 years] in the selected cities in the southern Ethiopia were considered as the study population.

### 2.2. Sample Size Determination and Sampling Procedure

The sample size was determined by using single population proportion formula the following assumptions: 95% confidence level, 5% margin of error, and taking 50% proportions (*p*).

(1) $$\begin{array}{c} n=\frac{{\left(z_{\alpha /2}\right)}^{2}*p\left(1-q\right)}{d^{2}}. \end{array}$$

Thus, the calculated sample size became 845 by considering two design effects due to the multistage nature of the study and 10% nonresponse.

Selecting seven cities: Arba Minch, Dilla, Durame, Hawassa, Mizan Aman, Soddo, and Yergalem among fourteen major cities in the southern Ethiopia lottery method was employed. To estimate number of street reproductive age groups in each selected city and the venues they frequently used for the purpose of survival work, an individual was assigned by an authorized person from the social and labor affair office. In addition, information regarding number of street-dwelling people in each city was requested from all concerned stakeholders. Finally, 845 samples were allocated proportionally to each selected cities. By using an individual who assigned by an authorized person, the first study respondent was identified. Then, the snowball sampling technique was used to recruit the representative sample [34].

### 2.3. Data Collection Tools and Procedure

Pretested and structured interviewer-administered questionnaire was used to collect the data. Data collection tool was adapted from the previous studies [1, 3, 6, 9–11, 16–18] and modified to fit the local context. The questionnaire designed to assess sociodemographic characteristics, alcohol use, and factors associated with alcohol use and sex after alcohol consumption. The pretest was conducted among 5% of street-dwelling people in nonselected cities. The result of the pretest used to modify the instrument. Four BSc midwives with previous experience on the data collection used as data collectors, and MSc holders were employed as supervisors.

### 2.4. Data Quality Control

The quality of the data was maintained by pretesting the tool and giving training for the data collectors and supervisors before the actual data collection. Every day after data collection, data reviewed and checked for completeness, accuracy, and clarity by the supervisors, and the necessary feedback was offered to data collectors. Data cleanup and crosschecking was done before analysis. To verify the internal consistency of the instrument, Cronbach's alpha analysis of alcohol use and sex after alcohol consumption-related questionnaire parts were done with the value of 0.81 which mean that the instrument items have good internal consistency.

### 2.5. Data Management and Analysis

The data were checked manually for completeness, coded, and entered into Epidata version 3.1 and exported to SPSS version 23.0 for analysis. Binary logistic regression was used to determine the association of independent variables with the outcome variables. Odds ratio with their 95% confidence interval and *P* value was used to identify the significant variables. The variables with *P* value less than or equal to 0.05 were considered as significant.

### 2.6. Operational Definition

Substance use: any substance that is taken into the living organism that may modify one or more of its function. In this study, the concept of substance use includes alcoholic drinks, tobacco, khat, and marijuana.

Alcohol use: street-dwelling people used alcoholic drinks such as beer and local alcohols [Tella and Xeji] during the previous one year of study either always, every day, or at least once a week reportedly were considered as alcohol use, while those who were not used at all and using less than once peer week were taken as not utilizing [29].

Sexual intercourse after taking alcohol: those respondents who had sexual intercourse after taking alcohols such as beer and local alcohols [Xeji and Tella] reportedly within one year prior to the study.

### 2.7. Ethical Consideration

Ethical approval was obtained from Hawassa University, College of Medicine and Health Sciences Ethical Review Board. Next, official letters were submitted to Regional Health Bureau. Letter of permission was obtained from Regional Health Bureau to Respective Zonal Health Department, Woreda Health Office, and City Administration. Furthermore, a written consent was obtained from Social and Labor Affairs Office. The written and verbal consent was obtained from each study subject prior to the data collection process proceeded. For those who were less than 18 years, written informed consent was obtained from their parent or guardian prior to the data collection process which was also approved by ethical review board [34].

## 3. Results

### 3.1. Sociodemographic Characteristics

A total of 842 street-dwelling people were included in the study with a response of 99. 6%. More than half 503 (59.7%) of them were males (Table 1).

### 3.2. Substance Use among Street Dwellers

More than half 423 (53.2%) of the respondents had taken a drink that contains alcohol (tella, tegi, areke, and beer) in the last one year of the study period. More than half, 265 (59%), of substance users ever had sex after taking alcohol in the last one year. Similarly, among the 324 khat chewers, 190 (58.64%) of the respondents ever had sex after chewing khat in the last one year (Table 2).

### 3.3. Alcohol Use and Cigarette Smoking

During the last 12 months of the study, 309 (36.7%) of the respondents had taken a drink that contains alcohol [CI: 35.5-39.7]. About 116 (13.8%) were drinking almost every day, and 193 (22.9%) drink at least once a week during the last one year. Regarding the frequency of alcohol use within the last 30 days of the study period, 155 (18.4%) of them did not remember, and 99 (11.8%) were taken every day reportedly.

More than one-third, 323(38.4%), of the street-dwelling people were smoking cigarette during the study period. Among the smokers, 114 (17.1%) were smoking every day, while 179 (21.3%) of them were smoking some days.

### 3.4. Factors Associated with Alcohol Use

Bivariate analysis factors found to be significantly associated with alcohol use were age, level of education, marital status, prior place of residence, and type of street, duration of stay on the street, ever heard about STDs, and being cigarette smokers. However, age, level of education, prior place of residence, ever heard about STDs, and being cigarette smokers were variables remain to be significantly associated with alcohol use in multivariate analyses. Accordingly, those who aged 19-25 and ≥26 years were about 51% (AOR: 0.49; CI: 0.34, 0.72) and 60% (AOR: 0.40; CI: 0.25, 0.65) less likely to use alcohol compared to those who were 15-18 years, respectively. Literate street-dwelling people were about 39% (AOR: 0.61; CI: 0.39, 0.94) less likely to use alcohol than those who were illiterate. In addition, those who were living in the major urban and small town prior to joining street life were about 69% (AOR: 0.31; CI: 0.18, 0.51) and 62% (AOR: 0.38; CI: 0.23, 0.63) less likely to use alcohol than those who were living in the rural area before joining street life, respectively. Likewise, those who ever heard about STDs were 86% (AOR: 0.14; CI: 0.07, 027) less likely use alcohol than their counterpart. Further, being a cigarette smoker were about 3 times (AOR: 2.67; CI: 1.94, 3.71) more likelihood of alcohol use compared to nonsmoker (Table 3).

### 3.5. Sexual Intercourse after Alcohol Use and Associated Factors

Out of the total 266 (62.9%) had sexual intercourse after taking alcohol within the last one year of the study period [CI: 58.2-67.4]. Bivariate analysis variables such as gender, age, educational status, type of street, history of rape, ever tested for HIV, and ever heard about STDs were significantly associated with having sexual intercourse after alcohol use. However, gender, educational status, history of rape, and ever heard about STDs were significantly associated with having sexual intercourse after alcohol use in multivariate analysis. Those who were females were about 80% (AOR: 0.20; CI: 0.12, 0.36) less likely to have sexual intercourse following alcohol consumption compared to those who were male. In addition, those who literate were also about 75% (AOR: 0.25; CI: 0.12, 0.51) less likely to have sexual intercourse following alcohol use compared to those who were illiterate. Furthermore, those who had a history of rape in the last one year of the study were about 5 times (AOR: 4.95; CI: 1.67, 14.65) more likely to have sexual intercourse following alcohol use than their counterpart. Those who ever heard about STDs were about 78% (AOR: 0.22; CI: 0.70, 0.68) less likely to have sexual intercourse following alcohol use than their counterpart (Table 4).

## 4. Discussion

During the last 12 months of the study period, 309 (36.7%) of the respondents had taken a drink that contains alcohol [CI: 34.8-40.7]. This finding is in line with the studies conducted in Texas (38%) [10], Toronto (40%) [22], and Egypt (35%) [23]. The present result is lower than the studies conducted in Los Angeles (30.8%) [19], Democratic Republic of Congo (63.5%) [24], and Ethiopia (64%) [18]; however, it is higher than the studies done in Ghana (12%) [6]. This discrepancy might be due to the difference in sociodemographic characteristics of the study respondents, sample size, and measurement tool. For instance, the study of Ghana was conducted among 227 street connected youths, and daily reported alcohol use was considered. In this study, those respondents reported used alcohol every day and at least once a week were considered as an alcohol user. This finding indicates the desire of an intervention on how to minimize substance misuses and its consequences among homeless people in the urban of Ethiopia.

In the present study, as the age increases, the consumption of alcohol decreases. This is in line with the previous study conducted in Ghana [6]. It could also be explained by the possible commencement of alcohol in early age which is viewed as a normal to create enjoyable physical and psychological environment [35]. In addition, literate street-dwelling people had also a less likelihood of alcohol use than those who were illiterate. This concept is also supported by the previous study done in Korea [36]. This might be due to the fact that being literate may increase a likelihood of awareness about the consequence of alcohol consumption. Another possible explanation is in the present study that more than two-thirds 655(77.8%) of the respondents had either elementary, high school, or college and above.

Place of residence prior to join street life was also significantly associated with alcohol use. Those who were living in the major urban and small town prior to join street life had less likelihood of alcohol use compared to those were living in the rural area. This might be due to the fact that those who joined street life from the towns might have awareness about the effect of alcohol use on health and sexual behaviors compared to those who joined street life from rural area.

On the other hand, ever heard about STDs was positively associated with alcohol use compared to their counterpart. This might be due to the impact of the shared information, and there might also an opportunity to hear about the health consequence of alcohol use. Moreover, being a cigarette smoker had also a more likelihood of alcohol use than nonsmoker. As evident in the previous studies, this is might be due to the intercorrelation between cigarette smoking and alcohol use [37, 38]. This finding is also in line with the previous study conducted among adolescents in Ethiopia [1].

Being a female had a less likelihood to practice sexual intercourse after alcohol use compared to those who were male. This might be due to the fear of sexual assault following alcohol consumption consequently resulted to unwanted pregnancy and its consequence. As evidenced by the previous study, females had a more likelihood of practicing safe sex than males [39] which could be achieved by avoiding sexual intercourse after alcohol use. The other possible explanations are because being a female is a protective factor of substance use [40].

Literate street-dwelling people had a less likelihood of sexual intercourse after alcohol use than those who were illiterate. This might be due to the fact that those who were literate can easily understand the adverse outcomes of sexual intercourse such as unwanted pregnancy and STDs. On the other hand, having history of rape prior to the study had a more likelihood of sexual intercourse after alcohol use than the counterpart. Even though there is no clear mechanism for the causality, this might be due to the unpleasant life events and psychological problems they faced during sexual assault which could made them to drink alcohol more and prone to sexual intercourse after consuming alcohol [41–43].

Moreover, the respondents who ever heard about STDs had also a less likelihood of having sexual intercourse after alcohol use than the counterpart. This might be due to the impact of the information gained about STDs and a fear of undesired sexual intercourse following alcohol use.

## 5. Limitations of the Study

Due to the nature of the study design, causality cannot be concluded. Moreover, as in all self-reported studies, we cannot rule out social desirability bias to sensitive questions, which might have introduced biases of unknown magnitude and direction.

## 6. Conclusions

In this study, significant percentage of respondents had taken a drink that contains alcohol one year prior to the study. Age, level of education, prior place of residence, ever heard about STDs, and being cigarette smokers were variables significantly associated with alcohol use. Furthermore, gender, educational status, history of rape, and ever heard about STDs were also significantly associated with having sexual intercourse after alcohol use. Awareness creation on the effect and outcome of substance use is recommended.

## Figures and Tables

**Table 1:** Sociodemographic characteristics of street dwellers in Southern Ethiopia, 2019 (*n* = 842).

Variables	Frequency	Percent
Gender		
Male	503	59.7
Female	339	40.3
Age in years		
15-18 years	246	29.3
19-25	407	48.3
≥26 years	189	22.4
Marital status		
Single/not ever married	521	61.9
Married	233	27.7
Divorced/separated	73	8.7
Widowed	15	1.8
Educational status		
Illiterate	187	22.2
Elementary	542	64.4
High school	102	12.1
College and above	11	1.3
Schooling status		
In school	97	11.5
Out of school	745	88.5
With whom currently living		
Alone	257	30.5
Peers	382	45.4
Boyfriend/girlfriend	135	16.0
Parents	50	5.9
Relatives	18	2.1
Place of current residence		
Hawassa	281	33.4
Soddo	165	19.6
Dilla	138	16.4
Arba Minch	117	13.9
Mizan Aman	108	12.8
Yergalem	20	2.4
Durame	13	1.5
Place of residence prior to starting street life		
Major urban town	313	37.2
Small town	369	43.8
Rural area	160	19.0
Type of street		
Street “on”	274	32.5
Street “off”	568	67.5
Duration of stay on the street		
< 5 years	621	73.8
5-10 years	168	20.0
> 10 years	36	4.3
Could not estimate	17	2.0
Daily income in birr		
5-20	136	16.2
21-50	518	61.5
≥ 51	654	77.7

**Table 2:** Substance abuse among street dwellers in Southern Ethiopia, 2019.

Variables	Frequency	Percent
A frequency of the drink contains alcohol use in the last 30 days (*n* = 842)		
Every day(30 days)	99	11.8
None in the last 30 days	56	6.7
Not sure/do not remember	155	18.4
A frequency of the drink contains alcohol use one year prior to the study (*n* = 423)		
Almost every day	116	13.8
At least once a week	193	22.9
Less than once a week	80	9.5
Not sure/do not remember	34	4.0
Sex after taking alcohol one year prior to the study		
Yes	265	31.5
No	155	18.4
Not sure/I do not know	1	0.1
Ever chewed khat one year prior to the study		
Yes	324	38.5
No	518	61.5
A frequency of chewing khat 30 days prior to the study		
Every day (30 days)	229	27.2
None in the last 30 days	36	4.3
Not sure/do not remember	59	7.0
Ever had sex after chewing khat		
Yes	190	22.6
No	127	15.1
Not sure/do not remember	1	0.1
A frequencies of smoking cigarette		
Every day	114	17.1
Some day	179	21.3
Not all	519	61.6

**Table 3:** Bivariate and multivariate analyses of factors associated with alcohol use among homeless reproductive age people in southern Ethiopia, 2019 (*n* = 842).

Variables	Alcohol use		OR (95% CI)	
	Yes	No	COR (95% CI)	AOR (95% CI)
Age in years				
15-18	71	175	1	1
19-25	153	254	0.67 (0.48, 0.95)	0.49 (0.34, 0.72)
26 and more	85	104	0.49 (0.33, 0.74)	0.40 (0.25, 0.65)
Educational status				
Illiterate	43	144	1	
Literate	266	389	0.48 (0.33, 0.70)	0.61 (0.39, 0.94)
Marital status				
Single	207	314	1	1
Married	60	173	1.90 (1.13, 2.68)	2.29 (0.82, 2.43)
Divorced	42	46	0.72 (0.46, 1.14)	1.22(0.73, 2.03)
Place of residence prior to joining street life				
Major urban town	136	177	0.31 (0.19, 0.49)	0.31 (0.18, 0.51)
Small town	142	227	0.38 (0.25, 0.59)	0.38 (0.23, 0.63)
Rural area	31	129	1	1
Type of street				
Street “on” [return to home at the night]	90	184	1.28 (0.95, 1.74)	1.27 (0.92, 1.77)
Street “off”[sleep on the street]	219	349	1	1
Duration of stay on the street				
< 5 years	194	427	2.66 (1.21, 4.36)	2.37 (0.89, 3.46)
5-10 years	86	82	1.15 (0.62, 2.14)	1.42 (0.73, 2.74)
> 10 years	29	24	1	1
Ever heard about STDs				
Yes	299	431	0.14 (0.07, 0.27)	0 0.14 (0.07, 027)
No	10	102	1	1
Cigarette smokers				
Yes	161	162	2.49 (1.87, 3.33)	2.67 (1.94, 3.71)
No	148	371	1	1

**Table 4:** Bivariate and multivariate analyses of factors associated with sexual intercourse after alcohol use among homeless reproductive age people in southern Ethiopia, 2019 (*n* = 842).

Variables	Sexual intercourse after alcohol use		OR (95% CI)	
	Yes	No	COR (95% CI)	AOR (95% CI)
Gender				
Male	126	129	1	1
Female	140	28	0.19 (0.12, 0.31)	0.20 (0.12, 0.36)
Age in years				
15-18	48	37	1.04 (0.59, 1.82)	1.06 (0.56, 1.98)
19-25	148	68	0.62 (0.39, 0. 98)	0.77 (0.46, 1.29)
26 and more	70	52	1	1
Educational status				
Illiterate	26	28	1	1
Literate	240	129	0.49 (0. 28, 0.89)	0.25 (0.12, 0.51)
Type of street				
^∗^Street “on”	70	52	1.39(0.90, 2.13)	1.70 (0.68, 2.78)
^∗∗^Street “off”	196	105	1	
Rape in the last 1 year				
Yes	61	4	11.38 (4.05, 31.98)	4.95 (1.67, 14.65)
No	205	153	1	1
Ever heard about STDs				
Yes	261	144	0.21 (0.07, 0.61)	0.22 (0.70, 0.68)
No	5	13	1	
Ever tested for HIV				
Yes	218	105	2.25 (1.43, 3.55)	1.37 (0.83, 2.26)
No	48	52	1	

∗ = Stay on the street at the day time and return to home at the night for sleep; ∗∗ = Stay on the street both during the day and night time.

## Data Availability

The data used to support the conclusions of this study are available from the corresponding author upon request.

## Abbreviations

STDs: Sexually transmitted diseases.
